# Agmatine modulates spontaneous activity in neurons of the rat medial habenular complex—a relevant mechanism in the pathophysiology and treatment of depression?

**DOI:** 10.1038/s41398-018-0254-z

**Published:** 2018-09-24

**Authors:** Torsten Weiss, René Bernard, Hans-Gert Bernstein, Rüdiger W. Veh, Gregor Laube

**Affiliations:** 1 Charité – Universitätsmedizin Berlin, corporate member of Freie Universität Berlin, Humboldt-Universität zu Berlin, and Berlin Institute of Health, Institute of Vegetative Anatomy, Berlin, Germany; 2 Charité – Universitätsmedizin Berlin, corporate member of Freie Universität Berlin, Humboldt-Universität zu Berlin, and Berlin Institute of Health, Klinik für Neurologie, Department of Experimental Neurology, Berlin, Germany; 3 Department of Psychiatry and Psychotherapy, Medical Faculty, University of Magdeburg, Magdeburg, Germany

## Abstract

The dorsal diencephalic conduction system connects limbic forebrain structures to monaminergic mesencephalic nuclei via a distinct relay station, the habenular complexes. Both habenular nuclei, the lateral as well as the medial nucleus, are considered to play a prominent role in mental disorders like major depression. Herein, we investigate the effect of the polyamine agmatine on the electrical activity of neurons within the medial habenula in rat. We present evidence that agmatine strongly decreases spontaneous action potential firing of medial habenular neurons by activating I1-type imidazoline receptors. Additionally, we compare the expression patterns of agmatinase, an enzyme capable of inactivating agmatine, in rat and human habenula. In the medial habenula of both species, agmatinase is similarly distributed and observed in neurons and, in particular, in distinct neuropil areas. The putative relevance of these findings in the context of depression is discussed. It is concluded that increased activity of the agmatinergic system in the medial habenula may strengthen midbrain dopaminergic activity. Consequently, the habenular–interpeduncular axis may be dysregulated in patients with major depression.

## Introduction

In the vertebrate brain, the medial habenular complex (MHb), together with the lateral habenular complex (LHb), is part of the epithalamus, an evolutionarily conserved brain region that is originally involved in the circadian regulation of biological rhythms^1^. During mammalian evolution, the habenula acquired additional functions, including in the context of anxiety and stress behavior, learning and memory, attention, and reward processing^2–4^. Thus, the habenula serves an integral part in the flow and processing of information from the so-called “limbic forebrain” to the midbrain^5,6^, thereby simultaneously influencing serotoninergic, dopaminergic, and cholinergic neurotransmitter systems. With respect to the morphology of afferent and efferent connections, however, the medial and lateral habenulae represent distinct entities^7^. The two nuclei are uni-directionally connected by axons originating in the MHb and projecting to the LHb^8^. The main input to the MHb via the stria medullaris arises from the triangular septal nucleus and septofimbral nucleus^6,9^. The predominant efferent projection of the MHb via the fasciculus retroflexus terminates in the interpeduncular nucleus (K, L), (IPN)^5,9^. The IPN, in turn, gives rise to ascending projections to limbic structures as well as descending projections to midbrain raphe nuclei, central gray, and dorsal tegmental nuclei^10^. Based on morphology, protein and mRNA expression, five subcomponents, namely the superior, inferior, central, lateral, and commissural subnuclei, have been initially described in the MHb^7,11^. More recently, the MHb has been conceived of as being subdivided into a dorsal component, essentially containing superior and dorsal subnuclei, and a ventral component, containing central, lateral, and medial subnuclei^12,13^. The different subnuclei project to different subdivisions of the IPN in a topographic manner^12,14^. Accordingly, the ventral IPN subnuclei (IPC and IPI) are selectively innervated by the medial part of the MHb, whereas the dorsal group of IP subnuclei is mainly innervated by the central and lateral MHb subnuclei^12^.

The MHb subnuclei use different combinations of neurotransmitter systems^11^. The superior part is glutamatergic and contains neurokinins, the dorsal part of the central MHb utilizes glutamate and substance P, while the ventral part of the central MHb as well as the inferior and lateral MHb are both cholinergic and glutamatergic. Furthermore, the MHb contains high concentrations of nicotinic acetylcholine receptors^15^ as well as GABA_B_ receptors^16,17^, the latter indicating the presence of strong GABAergic inputs^9^. The MHb–IPN axis is part of addiction pathways, most notably with participation in nicotine withdrawal and anxiety^18,19^.

With respect to dendritic and axonal morphology of MHb neurons, several different cell types have been described^8^. Despite their morphological differences, all MHb cells recorded in the aforementioned study shared the same electrical property, namely spontaneous tonic firing of action potentials at 1–10 Hz, which persisted after blocking of ionotropic glutamate and GABA_A_/GABA_B_ receptors. Without blocking synaptic transmission, massive AMPA/kainate receptor-mediated spontaneous excitatory postsynaptic potentials (EPSPs), most likely generated by neighboring MHb cells, were also evident, indicating the release of glutamate from at least a subpopulation of MHb neurons. According to a more recent study, cholinergic neurons account for the spontaneous action potential firing, whereas peptidergic neurons behave differently^20^. The temporal pattern of MHb cell firing can be modulated by a dual GABAergic synaptic response^21^. In contrast to the typical GABAergic inhibition, in the MHb there is a GABA_A_ receptor-mediated fast excitation^21,22^. It is followed by a slow inhibition due to GABA_B_ receptor activation as observed in rats at postnatal days 18–25. The GABAergic synaptic input is apparently exclusively extrinsic, because GABA-containing terminals, but neither GABA- nor GAD-containing cell bodies, were observed in the MHb (ref.^23^; own unpublished observation).

In the rat brain, agmatinase, an enzyme responsible for the inactivation of the putative neurotransmitter/neuromodulator agmatine (recently reviewed in ref.^24^), is prominently expressed in the MHb and the triangular septum^25^. Additionally, the expression of agmatinase is significantly upregulated in the MHb of depressed patients^26^ (→link to article). These observations led us to hypothesize, that the agmatinergic system may be a part of the information processing in the septal–habenular–interpeduncular axis. This postulation is supported by several further lines of evidence. Agmatine as well as the habenular systems are involved in depression^27–31^ and anxiety^18,32–35^. Although to date the LHb rather than the MHb has been discussed in the context of depression, there is accumulating evidence for an involvement for the MHb. Furthermore both, the LHb and the MHb, display structural abnormalities with respect to reduced volumes in patients suffering from affective disorders when compared to controls or patients with schizophrenia^36^. In contrast to the mainstream cortical theory of mood disorders^37^, a recent commentary by Loonen and Ivanova attempted to integrate the putative roles of LHb and MHb in depression and anxiety in subcortical forebrain and upper brainstem^38^.

Indeed, quite a number of experimental and clinical reports indicate that the cerebral serotonin system is critically involved in mood regulation. In this context, there is increasing evidence for the involvement of a dysbalanced serotonin system in the pathophysiology of depression^39,40^. Consequently, the question arises whether the involvement in depression is a direct effect of agmatine or rather mediated by the serotonergic system. In this context, Zomkowski et al. reported evidence for serotonin receptor involvement in an agmatine antidepressant like-effect in mouse^41^. However, Krass and colleagues demonstrated that the antidepressant-like effect of agmatine in the forced swimming test is not mediated by serotonin^42^.

In order to shed more light on the biological function of the agmatinergic system in the MHb, we have investigated the influence of agmatine and relevant imidazoline receptor (I1, I2) antagonists/agonists on spontaneous activity in neurons within the rat MHb. With regard to the roles of the agmatinergic and habenular systems in depression and anxiety, we extended our previous immunocytochemical studies on agmatinase protein expression in rat brain gaining additional insight into agmatinase expression in humans using post-mortem brains. The main goal of the present study was to obtain an electrophysiological evidence for a potential mechanism explaining the involvement of the agmatinergic system with psychiatric disorders, notably major depression. In order to extend our previous evidence for a dysregulation of the agmatinergic system in the MHb of depressed individuals^26^, we herein compared the distribution pattern for agmatinase within the rat and human MHb.

## Materials and methods

### Procedures of electrophysiological experiments

#### Slice preparation

Brain slices were prepared from 20 to 28 day postpartum (P20–28) male Wistar rats obtained from an institutional breeder (Forschungseinrichtungen für Experimentelle Medizin, Charite-Universitätsmedizin Berlin). Specimens were maintained under controlled temperature (22 °C) and scheduled illumination (12 h light/dark cycle) conditions with water and food ad libitum. All experiments were approved by the Regional Animals Ethics Committee (LaGeSo No.T 0127/02) and performed in strict accordance with the European Communities Council directive regarding care and use of animals for experimental procedures. All efforts were made to minimize the number of specimens and animal suffering. The animals were deeply anesthetized with isoflurane and then decapitated. Subsequently, the brains were dissected and transferred into 4 °C cold artificial cerebrospinal fluid (ACSF; see below). Coronal slices (400 µm thickness) containing the habenular complex were cut using a vibrating microtome (VT 1000S; Leica Instruments, Nussloch, Germany) and transferred into an interface-type recording chamber continuously perfused at a rate of 2.5–5.0 ml/min with prewarmed (34 °C) oxygenated ACSF containing in (mM): 125 NaCl, 25 NaHCO_3_, 2.5 KCl, 1.25 NaH_2_PO_4_, 2 CaCl_2_, 2 MgCl_2_, and 25 d-glucose, pH 7.4 maintained by saturation with carbogen (95% O_2_/5% CO_2_). In the ASCF solution used for brain preparation and during slicing, 50 mM sucrose was substituted for NaCl (to a final concentration of 75 mM). In addition, this solution contained (in mM): 0.1 CaCl_2_, 6 MgCl_2_, and 3 kynurenic acid (Sigma-Aldrich, now Merck; Merck KGaA, Darmstadt, Germany) to suppress transmission in the neuronal tissue. Before recording, the slices were allowed to recover within the recording solution (normal ACSF) for at least 1 h.

#### Electrophysiological recordings and data acquisition

Extracellular recordings were performed with glass micropipettes made from borosilicate glass tubing (outer diameter, 1.5 mm; wall thickness, 0.64 mm; Science Products, Hofheim, Germany) using a Sutter micropipette puller (P-97; Sutter Instruments, Novato, CA, USA). When filled with 0.5 M potassium acetate, pipettes resistances amounted to 5–10 MΩ. Single-unit activity was recorded using EXT-01C amplifiers (NPI Instruments, Tamm, Germany). Extracellular spikes were acquired with a CED 1401 plus interface (Cambridge Electronic Design Limited, Cambridge, UK) controlled by Spike2 software (CED Limited). The signals were digitized at 10 kHz and bandpass filtered at 0.3 and 3 kHz cutoff frequency. Once single-unit activity was achieved, spontaneous firing was monitored over 5–10 min to establish a stable baseline prior to the recording session.

#### Perfusion compounds (PC)

Brain slices were perfused with the following PC: agmatine sulfate (2 mM), moxonidine hydrochloride (100 µM), efaroxan hydrochloride (100 µM), and idazoxan hydrochloride (100 µM). All PC were purchased from Sigma-Aldrich (now Merck; Merck KGaA, Darmstadt, Germany). PC-containing solutions were freshly prepared prior to the experiments. They were diluted in ACSF from stock solutions dissolved in distilled water. Bath application was achieved via the perfusion system of the recording chamber.

#### Data analysis and statistics

Data analysis was done off-line using Spike2 (CED Limited) and Origin 6.0 (MicroCal, Northampton, MA, USA) software. Frequency histograms, number of spikes per second, mean firing rate, and percentage change from control (baseline) activity were determined for each experimental protocol. Each single-unit was evaluated for an increase or decrease in firing rate by comparing 15-min time intervals of activity (i) before pharmacological treatment (control), (ii) during PC administration, and (iii) after washout. For analysis, the time intervals were subdivided into 3-min bins. Experimental data were normalized to the initial basal firing rate for each single-unit. Basal firing was estimated as the frequency at the 3-min interval before treatment and taken as 100%. The PC bin interval containing the maximal change of frequency was reported as drug effect, whereas washout was estimated as the firing frequency within the very last interval of the respective washout phase. All numerical data are reported as mean ± S.E.M. To evaluate the statistical significance, data were subjected to Student’s *t*-test (Origin 6.0 software; Microcal, Northampton, MA, USA). A probability value of *P* ≤ 0.05 was considered to be significant.

### Histological and immunocytochemical techniques

All animal experiments were conducted in accordance with the guidelines of the European Communities Council directive 86/609/EEC and were approved by the Regional Berlin Animals Ethics Committee (LaGeSo No. G 0168/01). For immunocytochemistry, adult male Wistar rats were deeply anesthetized using a mixture of Ketavet (Parke-Davis, now Pfizer, New York, NY, USA) and Domitor (Pfizer). The animals were then perfused transcardially with 0.9% NaCl solution for 1 min followed by a fixative composed of 4% paraformaldehyde, 0.05% glutaraldehyde, and 0.2% picric acid for 20 min. For immunofluorescence, 4% paraformaldehyde only was used as a fixative. Brains were then removed from the skull. Tissue was rinsed extensively in 0.1 M phosphate buffer, and then freeze-protected with 1 M sucrose in 0.1 M phosphate buffer. Thereafter, tissue was frozen at −60 °C in hexane and stored frozen at −80 °C until use. For immunocytochemistry, a total of three rats and 90 frontal sections were analyzed. Immunocytochemical double-labeling experiments were performed 3–5 times for each combination of antigens.

#### Preparation of human tissue

All brains were obtained from pathologists or from medical examination officers, with the full consent of each family and in accordance with the ethics and rules outlined by German law and the local ethics commission of the University of Magdeburg. Brains of four individuals without neurological or psychiatric disorders (two males, 54 and 63 years; two females, 54 and 61 years) were studied. Brains were removed between 9 and 41 h after death. Tissue preparation was performed as described previously^43^. Briefly, brains were fixed in-toto in 8% phosphate-buffered formaldehyde (pH 7.0) for 2 months. After embedding the brains in Paraplast (McCormick Scientific, St. Louis, MO, USA), serial coronal 20-µm-thick sections were cut on a microtome and mounted on slides. Every 50th section was stained for morphological orientation (combined Cresyl Violet and myelin staining according to Nissl and Heidenhain-Woelcke).

#### Immunoperoxidase

Labeling was performed using standard diaminobenzidine/nickel-immunoperoxidase protocols as described previously^44^. Free-floating sections were treated with 1% sodium-borohydride in phosphate-buffered saline (PBS) for 15 min, washed with PBS, and incubated in a solution containing 10% normal goat serum (NGS), 0.3% Triton X-100, and 0.05% phenylhydrazine in PBS for 30 min. The characterization of the anti-agmatinase antibody was previously described^25^. It was diluted 1:1000 and 1:250, respectively, in 10% NGS, 0.3% Triton X-100 supplemented with 0.1% sodium azide and 0.01% thimerosal, and incubated for 36 h at 8 °C. Likewise, the anti-calretinin antibody (1:15,000, AB1550, raised in goat; Chemicon, Temecula, CA, USA) was applied.

After washing for 1 h in PBS and another hour in PBS containing 0.2% bovine serum albumin (PBS/BSA), the sections were incubated in biotinylated secondary goat anti-rabbit antibody (Vector, 1:2000 in PBS/BSA) for 24 h at 8 °C. The sections were again washed as described above and further incubated for 6 h with an avidin-biotinyl-peroxidase-complex (Vector Elite ABC kit) in PBS/BSA. After the final washing, bound peroxidase was visualized in a solution containing 1.4 mM diaminobenzidine, 10 mM imidazole, 6.6 mM nickel ammonium sulfate, and 0.15% H_2_O_2_ in 50 mM Tris/HCl buffer, pH 7.4. All sections were developed for 15 min. Labeled sections were mounted, dehydrated, and cover-slipped with Entellan.

#### Immunofluorescence

The incubation protocol used for immunofluorescence labeling (applies to supplemental figure S2) of rat brain tissue was identical with the immunoperoxidase protocol except for phenylhydrazine which was not used. Also normal donkey serum instead of NGS was used as a blocking reagent. Secondary antibodies used were goat anti-rabbit CY3, goat anti-mouse CY2, and donkey anti-goat CY2. Labeled sections were mounted on slides, air-dried, dipped in xylene, and cover-slipped with DPX. For double-labeling experiments, commercial antibodies against calretinin^45,46^ (1:15,000, AB1550, raised in goat; Chemicon, Temecula, CA, USA), parvalbumin^47^ (1:5000, P-3171, raised in mouse; Sigma, St Louis, MO, USA), calbindin^48^ (1:5000, C-8666, raised in mouse; Sigma), were used. For controls, either primary or secondary antibodies were omitted. No labeling was detected under these conditions.

#### Anterograde tracer injections

Two adult Wistar rats (264 and 275 g body weight) were anesthetized with 2–3% Isoflurane in 100% O_2_ and placed in a stereotactic frame (David Kopf, Tujunga, CA, USA). An isoflurane–O_2_ –mix was continuously delivered through a rat anesthesia mask (David Kopf). Core body temperature was monitored and maintained at 37 °C using a feedback‐controlled heating blanket. An access hole was made into the skull to unilaterally aim for the triangular septum according to a rat brain atlas. For the tracer injection, a glass pipette with end opening of 20 µm filled with 10% biotinylated dextran amine (BDA) was lowered into the brain at a 10° angle according to the following coordinates: Bregma −0.8 mm; lateral: +0.6 mm, distance from dura surface: −4.8 mm. BDA was ionotophoretically injected over 30 min. For pre- and post-injection, the electrode was maintained at position for 5 min. Finally, the pipette was removed from the brain and the scalp was closed with surgical sutures. Anesthesia was discontinued, and after recovery, the rats were transferred to their home cages. Three weeks later, the rats were deeply anesthetized by intraperitoneal injection of a mixture of 45 volume fractions ketamine hydrochloride (100 mg/ml; DeltaSelect, Pfullingen, Germany), 35 volume fractions xylazine hydrochloride (20 mg/ml; BayerVital), and 20 volume fraction saline 0.9%. This mixture was dispensed at a dose of 0.16 ml/100 g body weight. In addition, 200 IU heparin sodium (Heparin‐Rotexmedica, Trittau, Germany) were administered intraperitoneally to prevent blood clotting during perfusion. Anesthetized rats were transaortically perfused for 10 s with body-temperature warm plasma substitute (Deltadex 60; DeltaSelect), followed by 0.1 M phosphate buffer (pH 7.4) containing 4% paraformaldehyde, 0.05% glutaraldehyde, and 0.2% picric acid for 20 min. After a final 5‐min flush of the vasculature with 0.15 M sucrose in 0.1 M phosphate buffer (pH 7.4), the brains were removed, immersed in 0.8 M sucrose overnight for cryoprotection, sagittaly cut into blocks which were shock-frozen in hexane at −70 °C, and subsequently stored at −80 °C until they were serially cut into 25‐μm-thick sections. Thereafter, individual series were processed for light microscopic visualization of BDA. Free floating sections were washed in PBS containing 0.2% BSA (PBS-A) and 0.3% Triton, incubated overnight at 4 °C with peroxidase-labeled streptavidin (NEL 750; PerkinElmer, Rodgau-Jügesheim, Germany) diluted 1:20,000 in PBS-A. Next, sections were rinsed in PBS and pre-incubated in a solution containing 0.05% 3,3′-diaminobenzidine and 10 mM imidazole in Tris buffer (50 mM, pH 7.6). Next, ammonium nickel sulfite at 0.3% was added to the solution. The peroxidase reaction was started by the addition of hydrogen peroxide at a final concentration of 0.0015%. After 10–15 min under visual control, the reaction was stopped by repeated washes in PBS. Finally, sections were mounted onto gelatin-coated slides, dehydrated in a graded series of alcohol, cleared in xylene, and cover slipped. Digital images (applies to supplemental figure S4) of the slides were taking with an upright Leica DMRB light microscope connected to a high‐resolution digital camera (MBF‐CX9000; MBF Bioscience, Williston, VT). Images were adjusted for brightness and contrast in Adobe Photoshop CS3 and arranged in Adobe Illustrator CS3.

#### Retrograde tracer injections

Two further rats received microinjections aimed for the ventrolateral IPN/parainterfascicular nucleus of the VTA with the retrograde tracer gold-coupled wheatgerm agglutinin (WGA-apoHRP-gold; 15-nm particle size; 10–20 μg/ml in distilled H_2_O, pH 7.0–7.5; E-Y Laboratories, San Mateo, CA). Anesthesia, glass capillaries, and animal preparation were identical to the above-described tracer injections with the following stereotactic aim coordinates used: 5.8 mm posterior to bregma, 0.5 mm from the midline, and 8.0 mm ventral to the dura. Careful pressure application was used to inject approximately 0.3 µl tracer. The capillary was held in place for an additional 10 min before retrieving from the brain. After the wounds were closed and anesthesia discontinued, the animals were allowed to recover in their home cage. After 5 days, the animals were sacrificed, their brains were quickly removed, and they were processed for subsequent sectioning as described above (applies to supplemental figure S4). WGA-apoHRP-gold tracer was visualized in the section using a silver enhancement protocol^49^.

## Results

### Electrophysiology

**Fig. 1:**
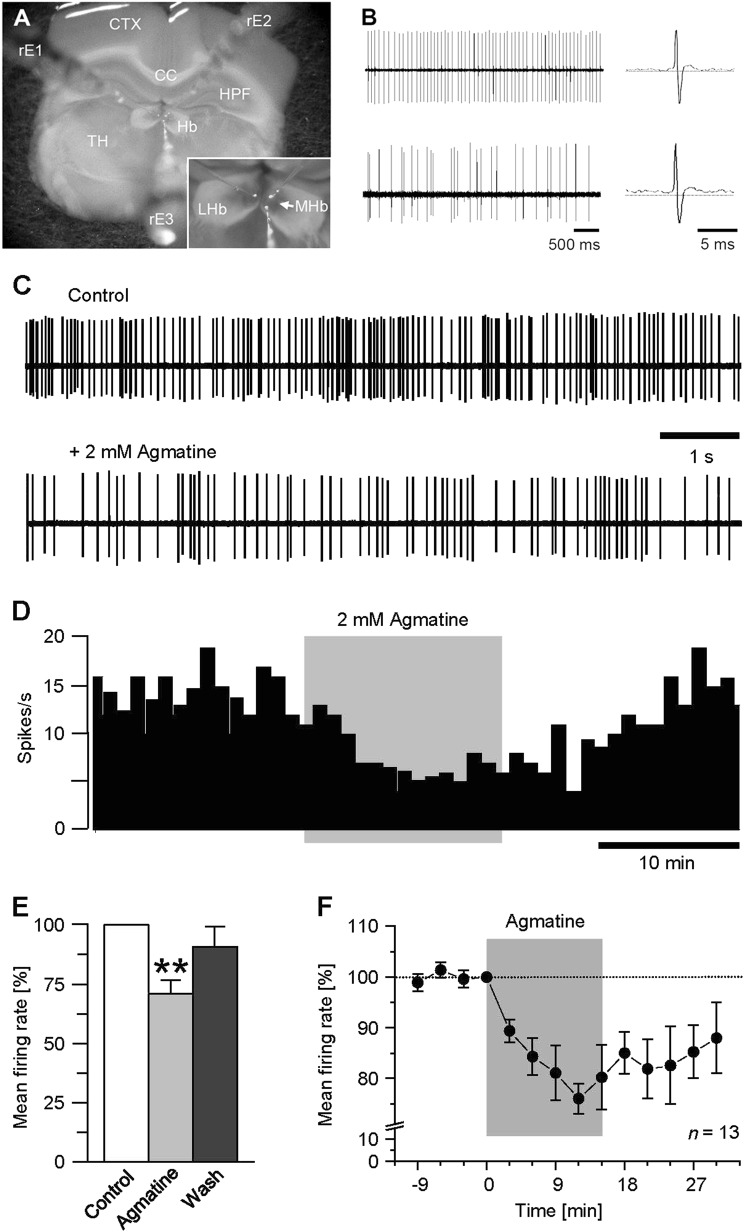
Agmatine suppresses spontaneous action potential firing within the rat MHb. **a** Photomicrograph of a coronal section (400-µm thick) of rat diencephalon comprising the habenular complex. Inset at higher magnification focuses on the arrangement of the recording electrodes for that triple recording. CC corpus callosum, CTX cortex, Hb habenular nucleus, HPF hippocampal formation, LHb lateral habenular complex, MHb medial habenular complex, rE1–3 recording electrodes, TH thalamic nucleus. **b** Examples of representative extracellular recordings from tonic regular (upper trace) and irregular (lower trace) firing MHb neurons. Expansions of the respective recordings shown at the right illustrate shape and time course of action potentials on enlarged time scales. **c** Representative recordings from another MHb neuron within a slice under control conditions (top trace) and with bath-applied agmatine (bottom trace). **d** Rate histogram (bin width 60 s) obtained from the experiment shown in *c* illustrating the agmatine-induced decrease in spontaneous action potential firing of the neuron. Note the recovering of discharge upon washout. **e** Bar graph summarizing the quantified data for the agmatine effect. Asterisk indicates a significance level of P ≤ 0.04 (*n* = 13). **f** Time course response diagram representing the results for the effect of agmatine on discharge frequency of 13 recorded MHb neurons. Normalized data are plotted as mean firing rate ± S.E.M. Note the discontinuity of *y*-axis. Gray backgrounds in *b* and *c* denote the time frame of agmatine administration

The physiological data presented in this study are based on extracellular single unit recordings obtained from 110 neurons within the MHb in slice preparations of 30 rats (Fig. 1a). MHb neurons displayed spontaneous discharge of action potentials at a mean firing rate of 9.5 ± 1.0 Hz in a range from 1 to 20 Hz. With respect to the pattern of discharge, MHb neurons both exhibited regular tonic firing of action potentials or repetitively discharged single action potentials randomly distributed (Fig. 1b, left panels). Characteristically, the recorded MHb neurons generated trains of broad (duration > 3 ms), triphasic extracellular spikes (Fig. 1b, right panels).

### Agmatine suppresses spontaneous action potential firing in rat MHb neurons

To investigate, whether the rat MHb underlies polyaminergic modulation, we tested the effects of agmatine on the basal firing rate of individual MHb neurons. Bath application of agmatine (2 mM) markedly suppressed spontaneous action potential firing of MHb neurons. A typical response is illustrated in Fig. 1c. Here, the discharge of the recorded neuron decreased from 15.1 spikes/s under control conditions to 5.2 spikes/s with agmatine (Fig. 1d). Similar results were obtained in another 12 neurons. In six neurons, agmatine slightly increased action potential firing, whereas in one recorded cell, neuronal activity was not altered. On average, agmatine decreased the mean firing rate of MHb neurons to 71.1 ± 5.5% of control (*P* *≤* 0.002, *n* = 13, Fig. 1e). The effect appeared within 1.5–3 min after application of the perfusion compound, reached a maximum at about 10 min after application, and was partially reversible with discharge recovering slowly upon washout within 30 min to 1.5 h (Fig. 1f).

**Fig. 2:**
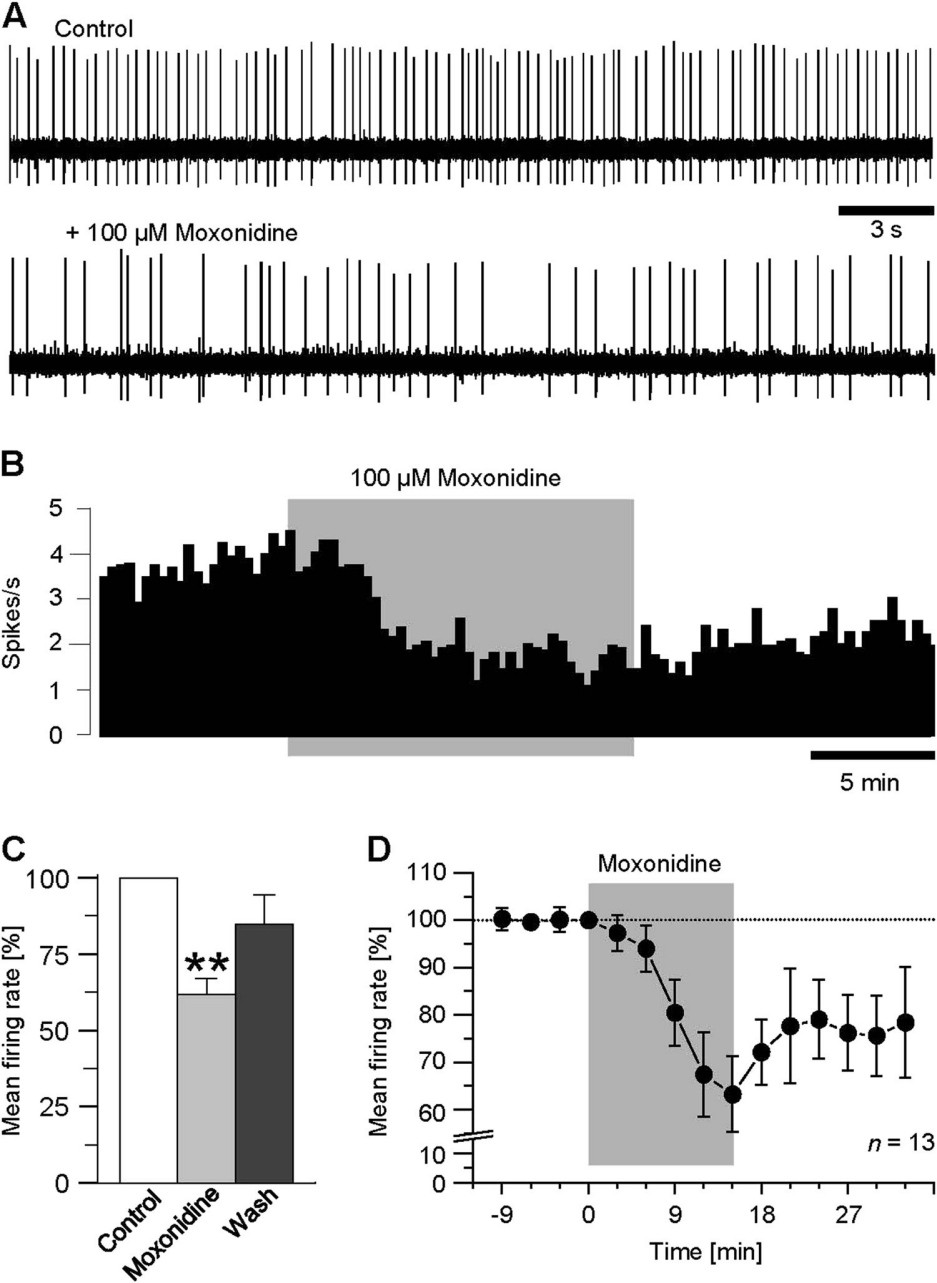
. Agmatinergic suppression of spontaneous action potential firing in the rat MHb is mimicked by the imidazoline-receptor agonist moxonidine. **a** Representative recordings from a MHb neuron under control conditions (top trace) and following bath application of moxonidine (lower trace). **b** Rate histogram (bin width 20 s) of the experiment illustrated in *a* showing the moxonidine-induced decrease of discharge in the recorded neuron. **c** Bar graph summarizing the results for the moxonidine effect. Asterisks indicate a significance level of P ≤ 0.01 (n = 13). **d** Time course response diagram illustrating the effect of moxonidine on MHb neuron firing obtained from 13 single-unit recordings. Normalized data are plotted as mean firing rate ± S.E.M. Note the discontinuity of y-axis

### Depression of MHb neuron discharge is mimicked by the imidazoline-receptor agonist moxonidine

To evaluate the biological relevance of the agmatine action onto spontaneous neuronal activity in rat MHb, we examined the influence of moxonidine, an agonist of endogenous imidazoline receptors. Not otherwise than agmatine, the imidazoline-receptor agonist distinctly suppressed action potential firing of MHb neurons (Fig. 2a). The quantification of the observed effect for the experiment illustrated in Fig. 2a revealed a decrease of discharge from 4.1 spikes/s under control conditions to 1.3 spikes/s with moxonidine (100 µM, Fig. 2b). Similar results were obtained in another 12 neurons. In three neurons, action potential firing was slightly enhanced. In total, the reduction of mean firing rate with moxonidine amounted to 61.8 ± 5.3% of control (*P* *≤* 0.001, *n* = 13, Fig. 2c). Although the agmatine-mediated reduction of spontaneous discharge was weaker (by approx. 29% with agmatine vs. 38% with moxonidine), the two effects were not significantly different (*P* = 0.14). As observed with agmatine, the moxonidine effect emerged within minutes after onset of treatment, reached a maximum at about 10 min after application, and was partially reversible upon prolonged washout (Fig. 2d).

**Fig. 3:**
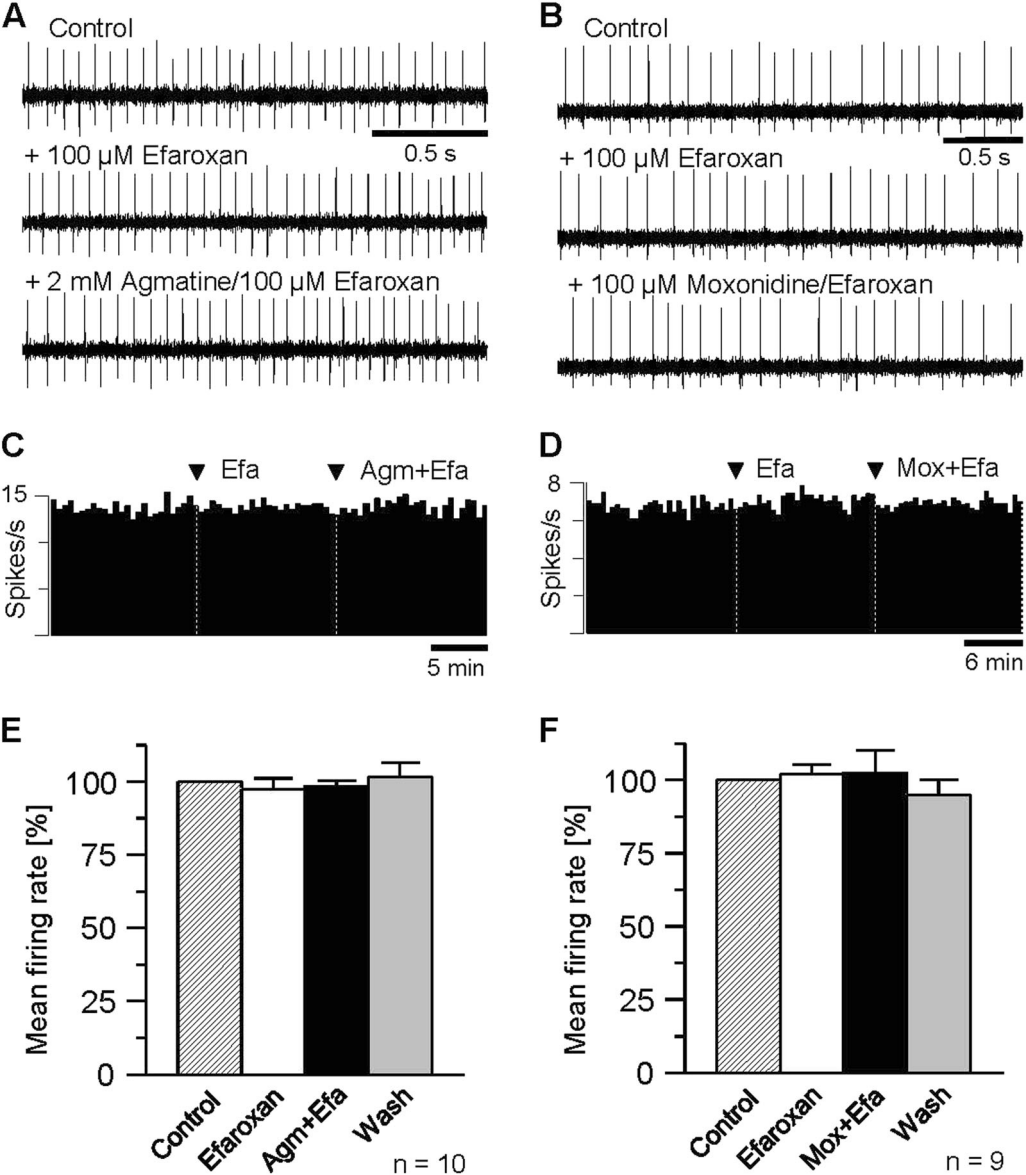
Agmatine- and moxonidine-mediated suppression of spontaneous discharge of MHb neurons is potently blocked by efaroxan, an I1-subtype imidazoline receptor antagonist. **a** Representative recordings from a MHb neuron in a slice under control conditions (top trace), following administration of efaroxan (middle trace) and upon efaroxan co-applied with agmatine (bottom trace). **b** Single-unit recordings from another slice under control conditions (top trace), following administration of efaroxan (middle trace) and upon efaroxan co-applied with moxonidine (bottom trace). **c** and **d** Rate histograms (bin widths 30 s) obtained from the recordings shown *a* and *b*, illustrating the responses with efaroxan alone and following co-application of the I1-receptor antagonist with agmatine and moxonidine, respectively. Arrow heads indicate the onsets of the respective application of the perfusion compounds. **e** and **f** Bar graphs summarizing the results of the experiments on efaroxan acting indicating a complete inhibition of both agmatine- (e, *n* = 10) and moxonidine- (f, *n* = 9) mediated suppression of action potential firing in the MHb. Normalized data are plotted as mean firing rate ± S.E.M

### Suppression of MHb neuron activity is mediated via I1-like imidazoline receptors

To elucidate the imidazoline receptor subtype involved in the depression of spontaneous action potential firing in MHb neurons, we examined the effects of selective antagonists of the imidazoline 1 (I1-like) and imidazoline 2 (I2-like) receptor, respectively.

First, we examined the influence of efaroxan, an inhibitor of the I1-like receptor on both agmatine- and moxonidine-mediated suppression of spontaneous discharge. As illustrated in Fig. 3, pretreatment (15 min) with the I1-antagonist efaroxan (100 µM) had no detectable effect on action potential firing of individual MHb neurons (Fig. 3a, b, middle traces). However, efaroxan strongly counteracted the depressant action of agmatine and moxonidine, respectively. Neither co-application of agmatine nor moxonidine with efaroxan altered MHb neuron discharge (Fig. 3a, b, lower traces). For the example shown in Fig. 3a, it is obvious from the respective time histogram that the firing rate of the recorded neuron was unchanged amounting to about 15 spikes/s under control, with efaroxan alone as well as with efaroxan and agmatine (Fig. 3c). Thus, efaroxan completely blocked the agmatine effect. Similar effects were observed in another nine MHb neurons. On average, mean firing rate after co-application of agmatine with efaroxan was slightly, but not significantly, decreased to 98.4 ± 2.3% of control (*P* = 0.439, *n* = 10, Fig. 3e), a value which did not differ from that obtained with efaroxan alone (97.4 ± 3.7%, *P* = 0.55) or after washout (98.6 ± 1.9%, *P* = 0.78), respectively.

**Fig. 4:**
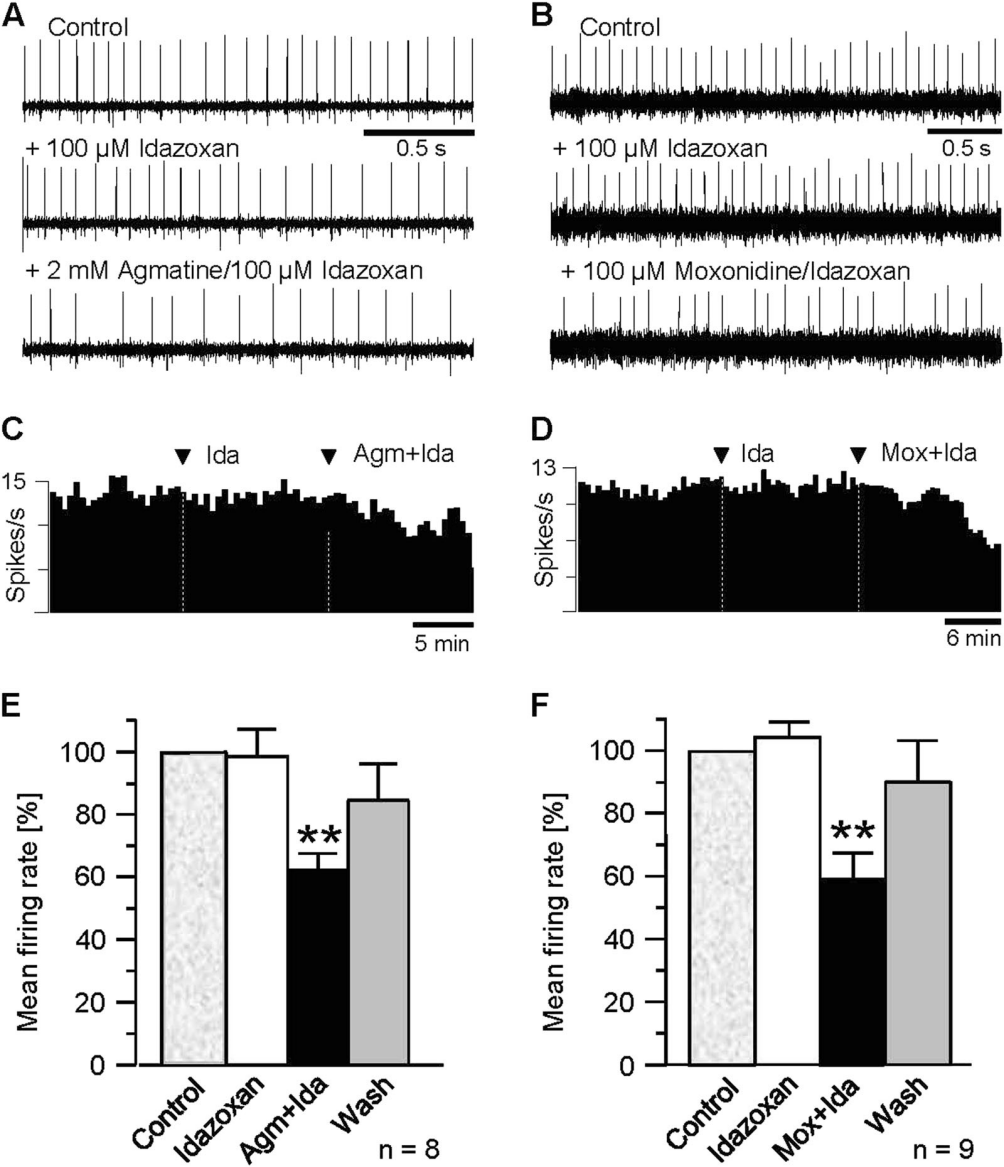
Agmatine- and moxonidine-mediated suppression of spontaneous discharge of MHb neurons is not altered by idazoxan, an I2-like imidazoline receptor antagonist. **a** Representative recordings from a MHb neuron in a slice under control conditions (top trace), with idazoxan (middle trace) and upon idazoxan co-applied with agmatine (bottom trace). **b** Single-unit recordings from another slice under control conditions (top trace), with idazoxan (middle trace) and upon idazoxan co-applied with moxonidine (bottom trace). **c** and **d** Rate histograms (bin widths 30 s) obtained from the recordings shown *a* and *b*, illustrating the responses with idazoxan alone and following co-application of the I2-receptor antagonist with agmatine and moxonidine, respectively. Arrow heads indicate the onsets of the respective application of the perfusion compounds. **e** and **f** Bar graphs summarizing the results of the experiments on idazoxan acting indicating the absence of any impact on both, agmatine- (**e**, *n* = 8) and moxonidine- (**f**, *n* = 9) mediated suppression of action potential firing in the MHb. Normalized data are plotted as mean firing rate ± S.E.M. Asterisks indicate a significance level of *P* ≤ 0.05 each

In the same way, efaroxan blocked the moxonidine-mediated suppression of action potential firing of MHb neurons. In the case of the recorded neuron illustrated in Fig. 3b, neither pretreatment with the I1-antagonist (100 µM) nor subsequent co-application of efaroxan and moxonidine altered spontaneous firing. Here, basal firing rate amounted to 7.3 spikes/s under control conditions, 7.6 spikes/s with efaroxan alone, and 7.3 spikes/s with co-application of efaroxan and moxonidine (Fig. 3d), indicating a complete inhibition of the moxonidine effect. Similar results were obtained in another eight recordings. On average, mean firing rate of MHb neurons during simultaneous treatment with efaroxan and moxonidine amounted to 102.3 ± 7.9% of control (*n* = 9, Fig. 3f). This value did not differ from that obtained with efaroxan alone (102.0 ± 3.2%, *P* = 0.53) and after washout (94.8 ± 5.2%, *P* = 0.084).

Secondly, we investigated the effect of idazoxan, an inhibitor of the I2-like receptor on agmatine- and moxonidine-mediated depression of action potential firing of neurons within the rat MHb. As observed for the I1-like antagonist efaroxan, pretreatment of slices with idazoxan (100 µM) had no detectable effects on spontaneous discharge of MHb neurons (Fig. 4a, b, middle traces). However, in contrast to efaroxan, the I2-antagonist did not prevent the suppressive effect of agmatine and moxonidine onto the firing rate of MHb neurons. Thus, as shown in Fig. 4a (lower trace), co-application of agmatine (2 mM) and idazoxan (100 µM) distinctly suppressed spontaneous discharge of individual MHb neurons. The quantification of the observed effect on the recorded neuron revealed a decrease of firing from 12.5 spikes/s under control conditions to 7.5 spikes/s with the two perfusion compounds (Fig. 4c). Similar results were obtained in another seven recordings. In three recordings, administration of idazoxan and agmatine slightly increased action potential firing. On average, the co-application of agmatine and idazoxan significantly reduced the mean firing rate to 62.2 ± 4.6% of control (*P* ≤ 0.003, *n* = 8, Fig. 4e), which is not different (*P* = 0.58) from the value observed with agmatine alone (71.1 ± 5.5%).

**Fig. 5:**
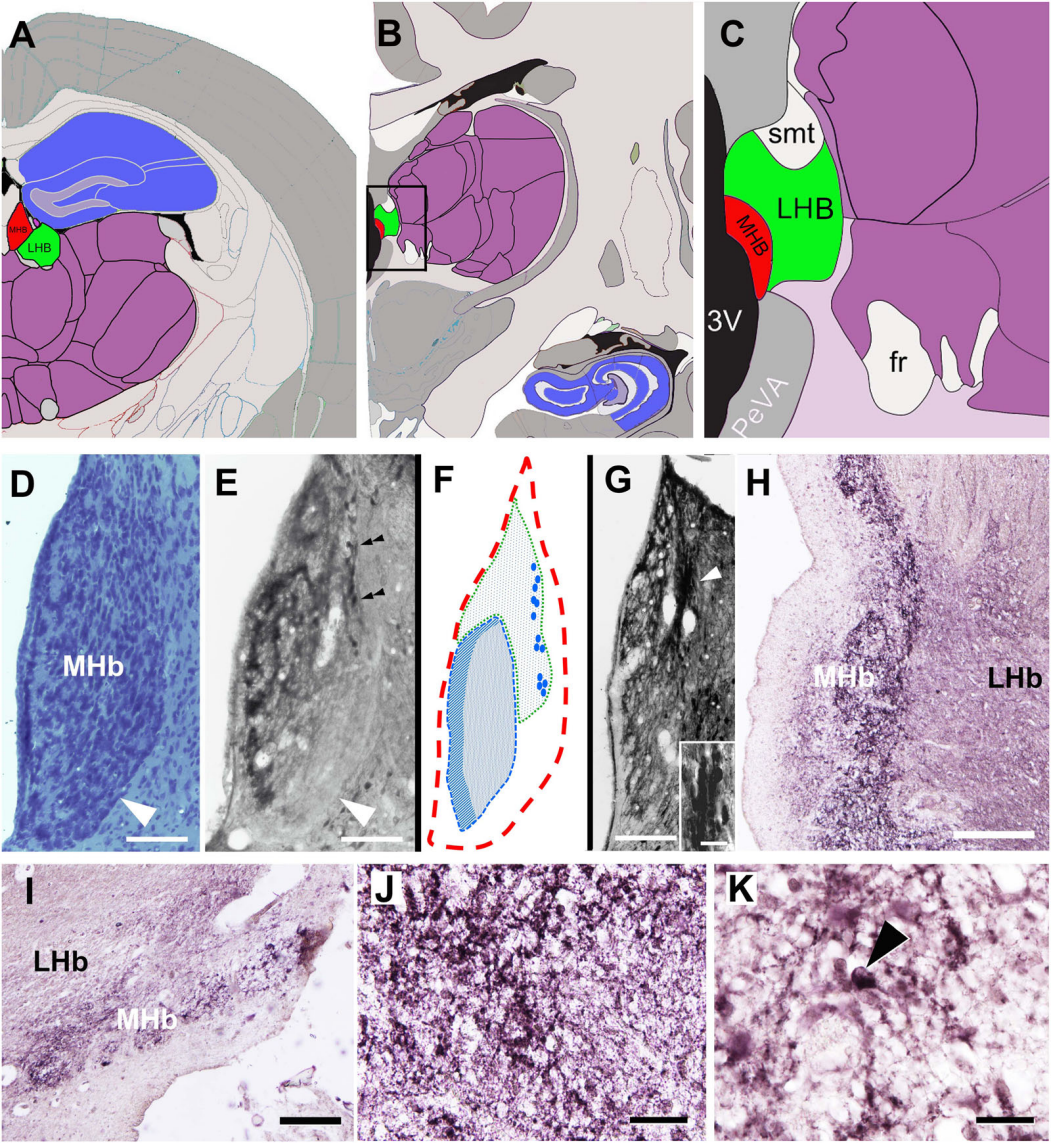
Agmatinase in the MHb. **a** Allen adult mouse brain reference atlas. **b** and **c** Allen adult human brain reference atlas^100^. Compared to the rodent MHb (**a**, shown in red), the human MHb (**b**, **c**) is located in a similar position, lining the lateral wall of the third ventricle, directly attached to the thalamus (purple). The relative position to the hippocampus (blue) is different due to the neocortical development in human brain. Image credit: Allen Institute (colors were modified). In cresyl violet-stained sections (**d**), the rat MHb appears relatively homogeneous and densely populated with neurons. Note the sharp border along the lower half of the nucleus (arrowhead). Agmatinase-like immunoreactivity (**e**) is mainly prominent in the neuropil, unveiling a sub-compartmentalization of the nucleus (ventrolateral border is indicated by an arrowhead). In addition, a stretch of strongly labeled neurons is evident in the upper lateral half (double arrowheads). The overall topography of neuropil and cell labeling is schematically delineated (**f**). With labeling for calretinin (**g**), a similar population of neurons (arrowhead and inset) is evident. The human MHb (**h**–**k**) also displays a heterogeneous agmatinase labeling pattern in frontal (**h**) and sagittal (**i**) sections, suggesting a similar sub-compartmentalization. A dense meshwork of fibers and terminals (**j**) forms a band of immunoreactivity clearly separated from the ventricular wall. Intensely labeled cell bodies are also evident (arrowhead in **k**). Scale bars = 100 µm in **d**, **e**, **g**; 20 µm in inset in **g**; 200 µm in **h**, **i**; 50 µm in **j**, **k**

Likewise, idazoxan failed to block the moxonidine effect on action potential firing of MHb neurons. A typical response to co-application of moxonidine and idazoxan is illustrated in Fig. 4b. In that neuron, the firing rate decreased from 10.3 spikes/s in control to 7.3 spikes/s with idazoxan and moxonidine (Fig. 4d). Similar effects were observed in another eight experiments. In four neurons, action potential firing was slightly enhanced. In total, the reduction of mean firing rate of MHb neurons with moxonidine and idazoxan amounted to 58.0 ± 9.1% of control (*P* ≤ 0.004, *n* = 9, Fig. 4f). This decline is not different (*P* = 0.79) from that obtained with moxonidine alone (61.8 ± 5.3% of control).

There is experimental evidence that beyond acting on imidazoline receptors, agmatine may interact with α2-adrenergic receptor binding sites^50^. Also the imidazoline receptor antagonists efaroxan and idazoxan have been shown to exert effects on that receptor type^51–53^. Therefore, we performed control experiments using yohimbine, a selective α2-adrenergic antagonist, to elucidate the possible involvement of α2-adrenergic receptors with the agmatinergic depression of action potential firing in the rat MHb. Our preliminary data indicate that an activation of α2-adrenergic receptors, in contrast to imidazoline I1 receptors, may only insignificantly account for the inhibition of action potential discharge induced by agmatine and moxonidine. This observation is in line with earlier investigations showing that (i) agmatine is about 30-fold selective for I1-, relative to α2-adrenoceptors^54^ and (ii) the high affinity component of platelet I1-binding sites displayed for agmatine a 800- to 5000-fold selectivity over the different types of α2-adrenoceptors^55^.

### Expression of the agmatinergic system in the MHb

The presence of agmatine in the neurons of the MHb has previously been shown^56^. However, the expression of agmatinase, one of the enzymes capable of inactivating agmatine in the brain^24^, was considered to reveal putative target areas of this neurotransmitter/neuromodulator in more detail^25^. Agmatinase was not only shown to be strongly expressed in rat but also in human MHb^25^. Thus, it may be speculated that similar patterns of activity with spontaneous firing of action potentials may also exist in the human brain. To date, however, this has not been experimentally verified. The similar differential distribution of agmatinase-positive fibers and neurons in both rat and human supports the notion of functional similarities. The overall morphological appearance of the habenula is similar in rat and human brain (Fig. 5a–c). However, due to differential neocortical development, the relative position of the habenula with respect to the hippocampus is remarkably different. In the rat, agmatinase-like immunoreactivity was localized to the neuropil and a subpopulation of habenular neurons located in the upper lateral aspect of the MHb (Fig. 5d, e). The neuropil labeling varied in intensity in distinct MHb areas. Accordingly, two prominent areas were detected along the ventricular border (schematically depicted in Fig. 5f).

### Co-localization with calcium-binding proteins

Calcium-binding proteins represent markers widely used to characterize neuronal populations^57^. When comparing agmatinase-like immunoreactivity with caldendrin (Fig. 5g), a calcium-binding protein which was previously reported to co-localize with agmatinase in other brain areas^25^, a similar stretch of immunopositive neurons was observed. Similar to the rat, the human MHb also displayed a prominent agmatinase-like immunoreactivity with distinctive subregions (Fig. 5h, k). The neuropil contained numerous labeled fibers and terminals (Fig. 5i). Intensely labeled cell bodies were also evident, but not as closely associated as in the rat (Fig. 5j).

In order to further characterize the neurochemical topography within the MHb, we employed a number of neurochemical markers displaying differential labeling patterns in this area (see supplementary Fig. S3). Among these markers, both calretinin and calbindin showed a pattern similar to the agmatinase distribution (Fig. S3 J,K,L). In addition to the MHb, this similarity in the distribution of the two calcium-binding proteins and agmatinase also applied for the LHb. Interestingly, both, the main input and output systems of the MHb, the triangular septum and the IPN, respectively, also prominently express agmatinase, as well as calretinin (Fig. S1 G-L). In order to further verify the overlap in the expression of agmatinase, calretinin, and calbindin in the same population of neurons, we employed immunofluorescence double labeling (Fig. S2). In addition to a co-localization of agmatinase and calretinin (Fig. S2 C, D), both the co-localization of agmatinase and calbindin (Fig. S2 A,B) as well as of calretinin and calbindin (Fig. S2 E,F) were observed in individual neurons. The connectivity of the MHb was previously analyzed^5,6,14^. We re-evaluated this network with anterograde and retrograde labeling (see Fig. S4).

## Discussion

In the epithalamus, the habenulae represent the relay station connecting limbic forebrain structures with midbrain diffuse neurotransmitter systems, including the dopaminergic and serotoninergic systems. Within this relay station, the MHb and the LHb form separate entities with regard to morphology, connectivity, and electrical activity. Both nuclei, however, consist of several subnuclei, indicating further complexity of the involved circuits. Despite this morphological and neurochemical heterogeneity, neurons within either the LHb on one hand and the MHb, on the other, are characterized by similar patterns of electrical activity. While lateral habenular neurons produce action potentials in burst mode upon activation, a significant fraction of MHb neurons spontaneously generates tonic trains of action potentials^8,58^. When compared with previous studies^8,59^, the mean frequency of action potential trains measured in this study, however, was obviously higher [9.5 Hz (1–20 Hz) as compared to 5.0 Hz (1–10 Hz; ref.^8^) and 4.2 Hz (2–10 Hz; ref.^59^)]. The latter study also revealed that cholinergic, but not peptidergic, neurons show spontaneous action potential firing.

With respect to neurotransmitter release, the dorsal component of the MHb was shown to express substance P^60^, while the ventral component accounts for acetylcholine release in the IPN^61^. In addition to acetylcholine and substance P, glutamate is also released by MHb projection neurons in the IPN^14^. Glutamate, in addition to ATP, is also released in the MHb by axons arising from the triangular septum^14^. Furthermore, genetic ablation of MHb neurons led to a massive reduction of acetylcholine in the IPN and a variety of behavioral abnormalities^62^. Thus, any interference with cholinergic and glutamatergic signaling in the dorsal diencephalic conduction system can be expected to significantly influence the balance of limbic circuits. Agmatine is known to interact with both, cholinergic and glutamatergic, systems. It blocks exocytosis in chromaffin cells by blocking nicotinic acetylcholine receptor currents^63^ and is involved with nicotine^64^, morphine^65^, and ethanol^66^ withdrawal. With respect to glutamate receptors, agmatine has been shown to antagonize the NMDA subclass in a concentration- and voltage-dependent manner in the hippocampal neurons^67^. Hence, the aforementioned withdrawal syndromes may be related to glutamate signaling at NMDA-receptor-containing synapses (reviewed in refs.^24,68^). In the present study, we show that agmatine attenuates the tonic firing of action potentials in the MHb. Since the IPN is the major target of the MHb and given that the IPN activity in turn decreases the midbrain dopamine release^69^, it can be concluded that agmatine may regulate the inhibitory effect of the habenula–interpeduncular pathway on the reward system. This idea is further supported by the fact that agmatine seemingly acts as an endogenous anti-depressant^70^ and a malfunction of the reward system is a hallmark of depression^71–73^.

Since agmatine may interact with different receptor types, including α2-adrenergic and imidazoline receptors, we tested different agonists and antagonists in order to characterize the nature of the suppression of tonic activity in the MHb. Accordingly, the inhibitory effect is apparently mediated by activation of imidazoline receptors of the I1-subtype. Both imidazoline I1 and I2 receptor subtypes have been reported to be expressed in neurons and astrocytes^74^. In the MHb, an imidazoline receptor protein was immunocytochemically localized^75^. Also, [3H]-RS-45041-190, a selective high-affinity radio ligand for I2 imidazoline receptors, intensely labeled the MHb^76^. Moreover, imidazoline I1 receptors (IRAS/nischarin; see: © 2017 Allen Institute for Brain Science. Allen Mouse Brain Atlas. Available from: http://mouse.brain-map.org/, ISH experiment 69134550, P56 mouse, section no. 18, accessed at 04/27/2018 12:50 pm) and imidazoline I2 receptors^77^ have both been localized to the MHb by in situ hybridization. Furthermore, clonidine, an imidazoline and α2-adrenergic agonist^78,79^, was shown to attenuate increased brain glucose metabolism during naloxone-precipitated morphine withdrawal in the MHb^80,81^. Similarly, agmatine prevented the naloxone-precipitated abstinence syndrome in morphine-dependent rats^65^. Thus, a role for agmatine acting via imidazoline I1 receptors in addiction, as well as possibly in depression, may be hypothesized (reviewed in ref.^24^). In support of our data, the involvement of I1 imidazoline receptors with the antidepressant-like action of agmatine in mouse models of depression has been elegantly demonstrated using different behavioral tests^82^. Interestingly, the mouse I1 receptor homolog IRAS/nischarin was found to be downregulated in the prefrontal cortex of postmortem brains from individuals with major depression who received antidepressant drug treatment^83^. In subcortical limbic structures, however, this may be different. In this context, the role of the MHb-IPN-pathway in addiction and mood regulation has been recently reviewed^84^.

With regard to the subnuclear organization of the MHb, agmatinase-like immunoreactivity is found in a subpopulation of neurons in the dorsolateral aspect and diffusely throughout the neuropil of the nucleus, but most prominently in the ventromedial aspect (Fig. 5f). According to our data from retrograde tracing in the rat IPN (Fig. S4), the dorsal half of the MHb is apparently connected to the lateral IPN, which in turn also contains the most intense immunosignal for agmatinase within the IPN (Fig. S1l). On the other hand, the ventral half of the MHb is highly enriched with nicotinic acetylcholine receptors^85^. For comparison, other markers like L-enkephalin, NK-A, and calbindin (see supplemental figure 1) define areas within the MHb only partially overlapping with the peptidergic dorsal and the cholinergic ventral region. Thus, a revision of the originally morphologically defined subnuclei of the MHb may need a systematic effort in order to integrate all known data regarding putative functional subdomains. A recent transcriptomic–anatomic study supports the idea of the presence of novel subfields^86^.

The etiology of major depressive disorder is undoubtedly complex and several pathophysiological mechanisms have been intensely examined, including the monoamine hypothesis, the glutamate hypothesis, genetic factors, endocrine factors (notably the Hypothalamus-Pituitary-Adrenal axis dysfunction), environmental stress factors, and immunologic factors (reviewed in refs.^87,88^). However, common therapies are frequently based on the interference with monoamine levels by using monoamine oxidase inhibitors and serotonin re-uptake inhibitors. Interestingly, a robust expression of the serotonin receptor 5HT-4 was observed in the lower half of the MHb (5 hydroxytryptamine receptor 4; see: © 2017 Allen Institute for Brain Science. Allen Mouse Brain Atlas. Available from: http://mouse.brain-map.org/, ISH experiment 72119658, P56 mouse, section no. 29, accessed at 07/23/2018 12:43 pm). This expression pattern is at least partially overlapping with the most prominent labeling for agmatinase within the MHb. In concordance, in the hamster brain, a dense innervation of the MHb by 5-HT-IR fibers has been shown, in contrast to an only very-sparse innervation from the dorsal and median raphe nuclei^89^. Thus, it is tempting to speculate that therapy with serotonin re-uptake inhibitors may interfere with the agmatinergic system in this area. Noteworthy, also monoamine oxidase is robustly expressed in the lateral part of the MHb (MAO A; see: © 2017 Allen Institute for Brain Science. Allen Mouse Brain Atlas. Available from: http://mouse.brain-map.org/, ISH experiment 74750015, P56 mouse, section no. 27, accessed at 07/25/2018 10:15 pm) and may be inhibited by agmatine under certain conditions^90^. In addition to serotonin and norepinephrine, a diminished dopaminergic neurotransmission and an impaired cholinergic transmission are evidently involved in the pathophysiology of major depression and of addiction^91,92^. To this end, the habenula apparently acts as a central regulator of diffuse neurotransmitter systems. While the LHb modulates serotonin levels and norepinephrine release, the MHb regulates acetylcholine and dopamine levels^93,94^. Given that in congenitally helpless rats, an animal model for depression, an elevated metabolism of 64–71% in the habenula and of 25% in the interpeduncular nucleus (K, L), were observed^31^, the habenular–interpeduncular axis seems to represent a promising target for the development of new therapeutic strategies. In this scenario, the results presented here suggest that the pharmacologic interference with the agmatinergic system may promise great potential in order to develop related strategies.

## Conclusion

In summary, we conclude that our electrophysiological data support the idea that the agmatinergic system influences brain function in addiction, anxiety, and depression. Accordingly, the release of agmatine in the MHb, acting on imidazoline I1 receptors, would lead to decreased excitation of the IPN and hence to an increase of dopaminergic function in the midbrain. Besides the relevance for depression, for more detailed information on the role of agmatine in addiction and anxiety, we refer to recent reviews^24,68^. Interestingly, anxiety increased the activity of neurons in the IPN^95^. This activation during nicotine withdrawal was mediated by increased corticotropin releasing factor receptor-1 expression and signaling, which in turn modulated glutamatergic input from the MHb. Thus, there is evidence that both agmatine and the MHb may play pivotal roles not only in depression but also in anxiety and addiction. Similar to the LHb, the MHb function probably has to be expected to fall into several subsystems. The habenula on the whole likely represents a bottleneck in the subcortical emotional brain circuits. Consequently, deep brain stimulation in this area has undergone preclinical and clinical trials^96^ (reviewed in refs.^97–99^). Interference with the agmatinergic system, however, may offer a pharmacological alternative to surgical therapy. Currently, our electrophysiological experiments are limited to normal (“not depressed”) experimental animals. In the future, it will be interesting to gain additional insight with similar experiments performed with brains derived from animal models for depression, like congenital helpless rats.

## Electronic supplementary material

Supplementary figure legends

Supplementary figure 1

Supplementary figure 2

Supplementary figure 3

Supplementary figure 4
