# Supplementary material for: Agmatine modulates spontaneous activity in neurons of the rat medial habenular complex—a relevant mechanism in the pathophysiology and treatment of depression?
Source: Transl Psychiatry. 2018 Sep 24;8:201. doi: 10.1038/s41398-018-0254-z (PMC6155246; doi:10.1038/s41398-018-0254-z)
Supplement: Supplementary file 1 — Supplementary figure legends [file 41398_2018_254_MOESM1_ESM.docx]

**Supplementary figure legends:**

**Fig. S1.** Agmatinase and calcium binding proteins in the MHb, the septum, and the interpeduncular nucleus. Agmatinase (**B**), calretinin (**A**), and calbindin (**C**) are all expressed in the medial (MHb) as well as the lateral (LHb) habenula. Overall the patterns of immunoreactivity are all similar, especially with respect to a group of small neurons (arrowheads). In the corresponding enlarged micrographs (**D-F**) the arrowhead points at identical positions within the parallel sections. In the triangular septum (**G-J**) both, agmatinase and calretinin are intensely expressed in neurons (**I, J**) throughout the area. In the interpeduncular nucleus **(K, L)**, neuropil as well as neurons are labelled for both antigens, however, most intense labelling is evident in the lateral (IPL) and dorsolateral (IPDL) subnuclei when compared to the central (IPC) and rostral (IPR) subnuclei. Antibodies: calretinin, 1:15,000, AB1550, raised in goat; Chemicon,

Temecula, CA, USA; calbindin, 1:5,000, C-8666, raised in mouse; Sigma-Aldrich. Scale bars = 200 µm in A-C, K, L; 500 µm in G, H; 100 µm in I, J; 25 µm in D-F.

**Fig. S2.** Immunofluorescence double labelling for agmatinase, calbindin, and calretinin. In the dorsolateral aspect of the MHb, agmatinase (**A** and **C**) is expressed in calbindin (**B**) as well as calretinin (**D**) containing neurons. Compare with the labelled cells in Fig. S1. In the same area, also neurons containing both, calretinin and calbindin were observed (**E**, **F**). Antibodies: calretinin, 1:15,000, AB1550, raised in goat; Chemicon, Temecula, CA, USA; calbindin, 1:5,000, C-8666, raised in mouse; Sigma-Aldrich. Scale bar = 100 µm.

**Fig. S3**. Immunocytochemical markers in the MHb. Rostrocaudal serial sections were labelled for cresyl violet (**A-C**), leu-enkephalin (**D-F**), neurokinin A (**G-I**), calbindin (**J-L**), choline acetyl transferase (**M-O**). Antibodies: leu-enkephalin, 1:20,000, ICH 8601, raised in rabbit, Peninsula Lab, Bachem; neurokinin A, 1:1,000, ICH 7359, raised in rabbit, Peninsula Lab, Bachem; calbindin 1:5,000, C-8666, raised in mouse; Sigma-Aldrich; choline acetyl transferase, 1:2,000, C-2888, Sigma-Aldrich. Scale bar = 200 µm.

**Fig. S4.** Anterograde and retrograde ­­tracing of the MHb. (**A**) Allen Adult Mouse brain reference atlas. Sagittal section depicting shape, location and boundaries of triangular septum (TRS), medial habenula (MHb) and lateral habenula (LHb). (**B** and **C)** BDA anterograde tracer injected in the triangular septum (TRS) labels axonal terminals in the MHb but not LHb. (**D)** The traced axonal path between TRS and MHb runs along the ventral aspect of the stria medullaris (arrowheads). (**E)** WGA retrograde tracer injected at the lateral border of the IPN. The injection path is marked by the arrowhead. (**F)** The WGA tracer is transported to neurons located in the superior half of the MHb.
