# Supplementary figures and images for: Agmatine modulates spontaneous activity in neurons of the rat medial habenular complex—a relevant mechanism in the pathophysiology and treatment of depression?
Source: Transl Psychiatry. 2018 Sep 24;8:201. doi: 10.1038/s41398-018-0254-z (PMC6155246; doi:10.1038/s41398-018-0254-z)

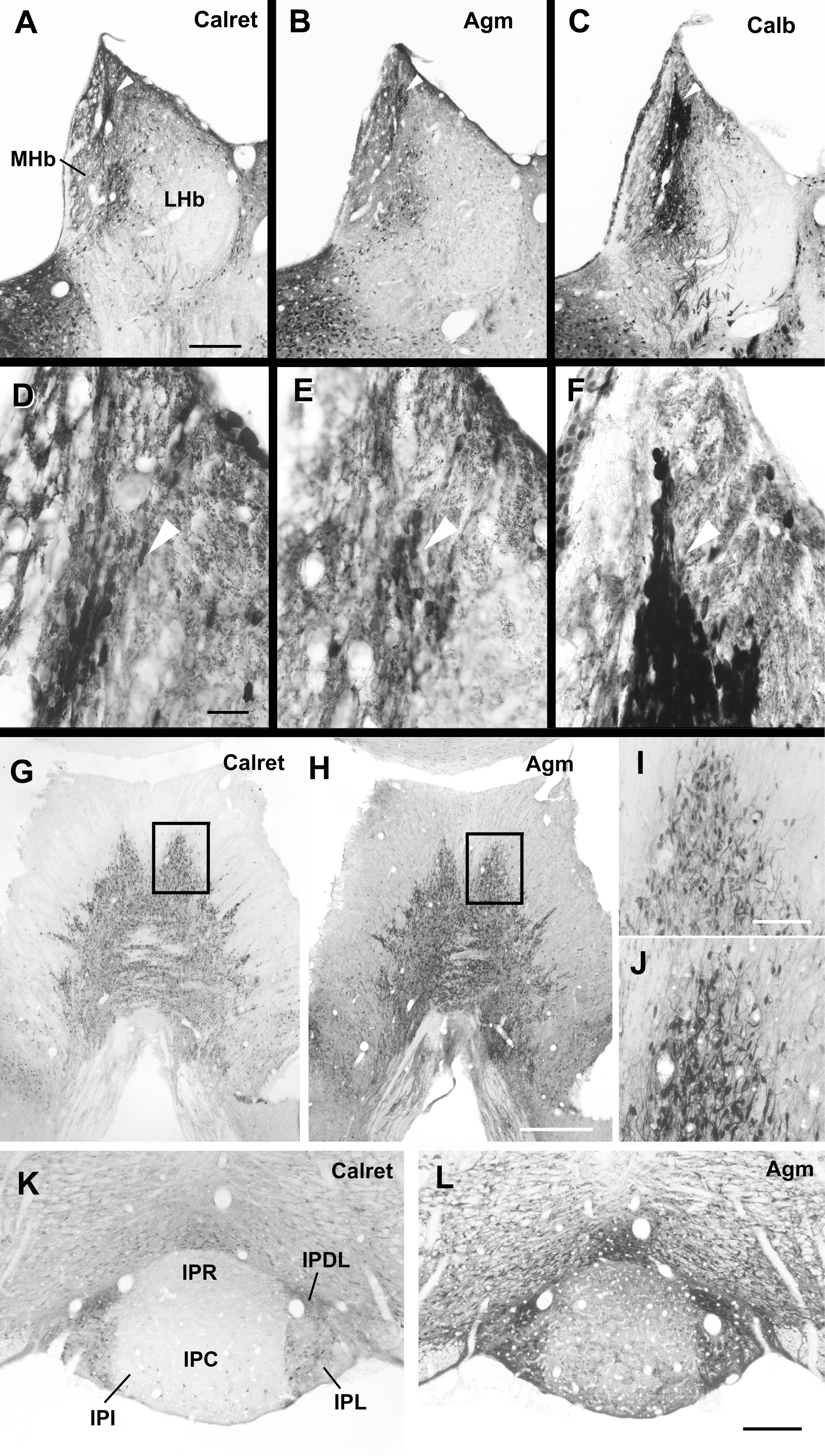

Supplement: Supplementary file 2 — Supplementary figure 1 [file 41398_2018_254_MOESM2_ESM.jpg]

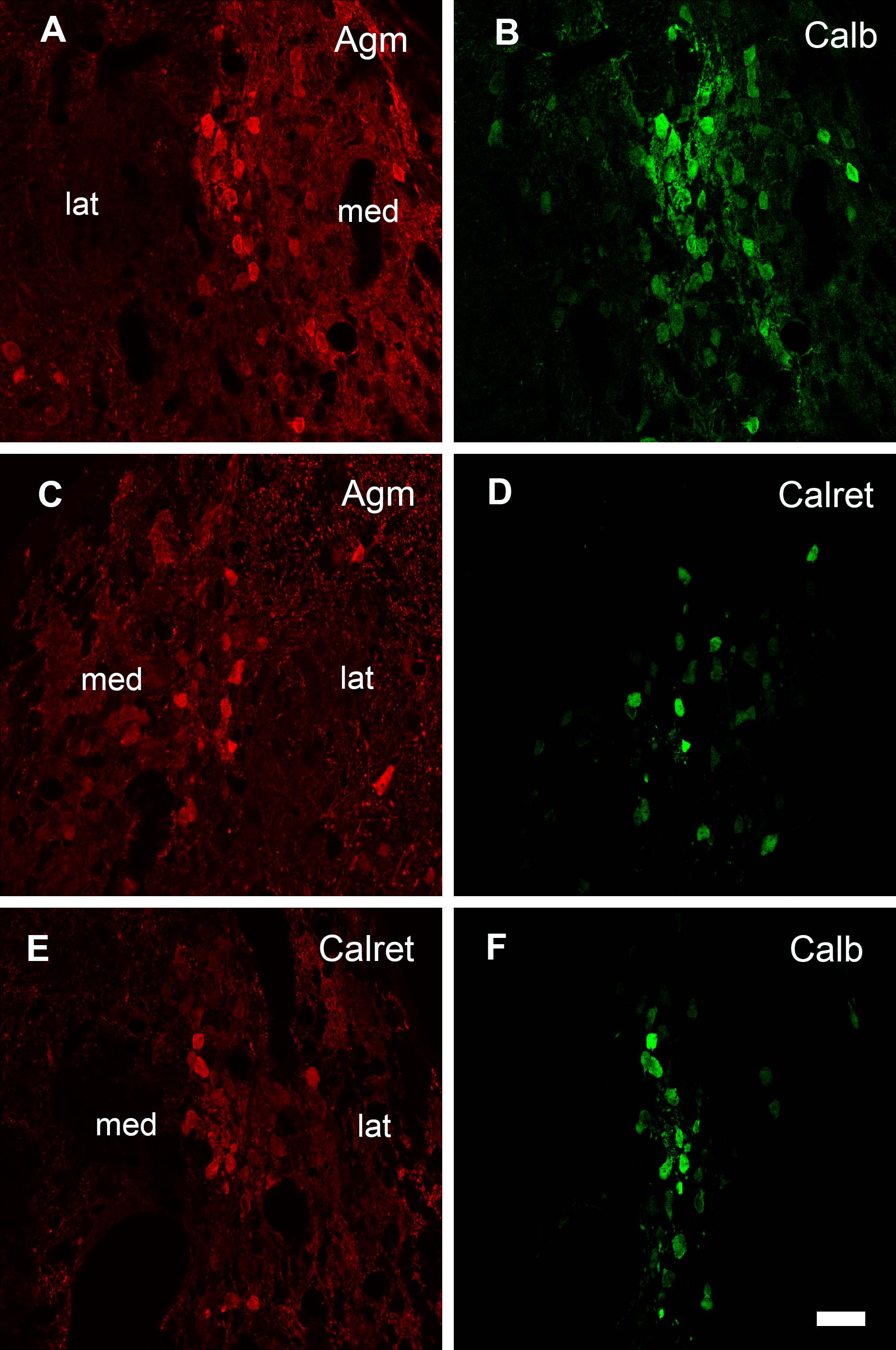

Supplement: Supplementary file 3 — Supplementary figure 2 [file 41398_2018_254_MOESM3_ESM.jpg]

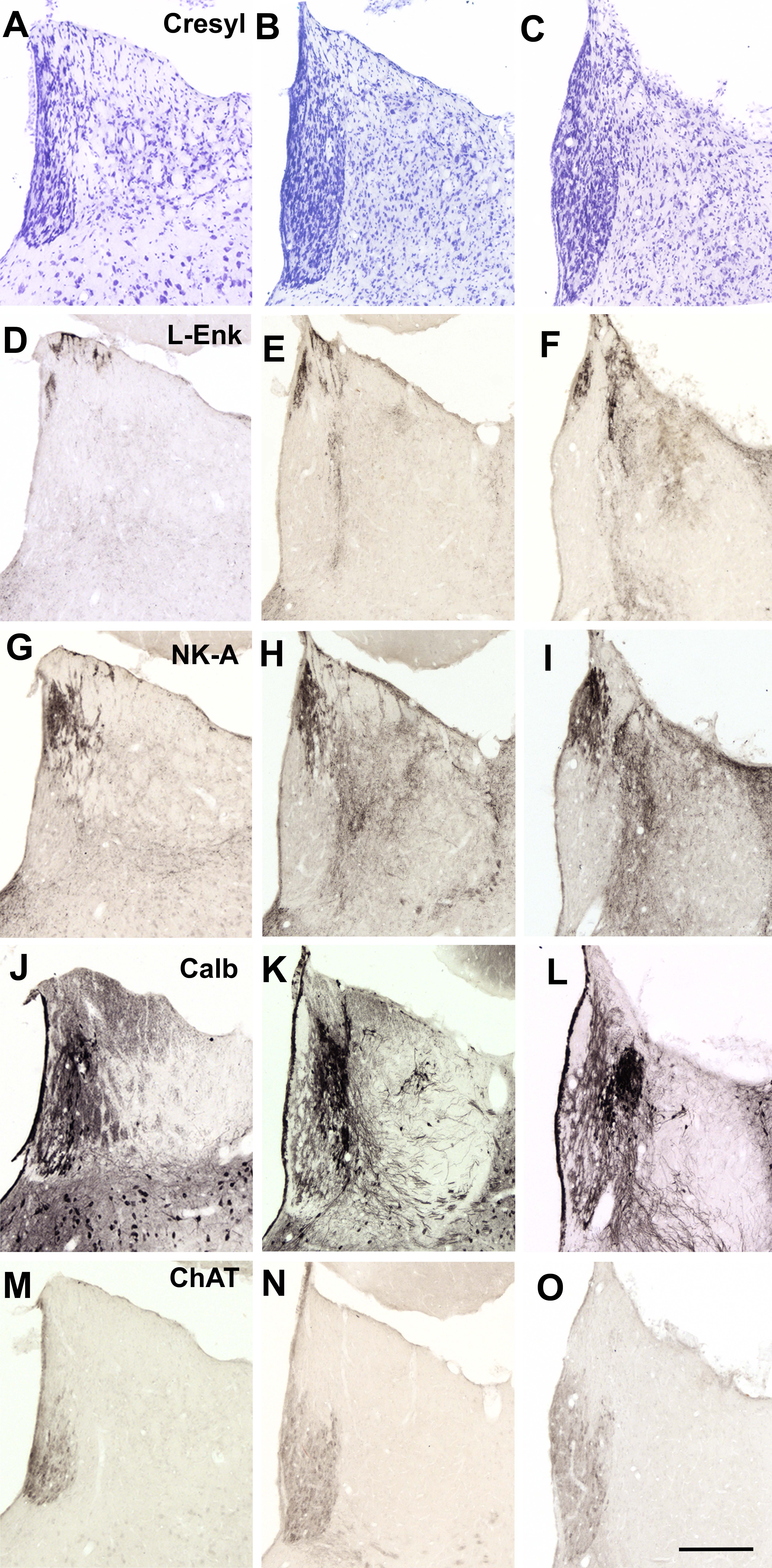

Supplement: Supplementary file 4 — Supplementary figure 3 [file 41398_2018_254_MOESM4_ESM.jpg]

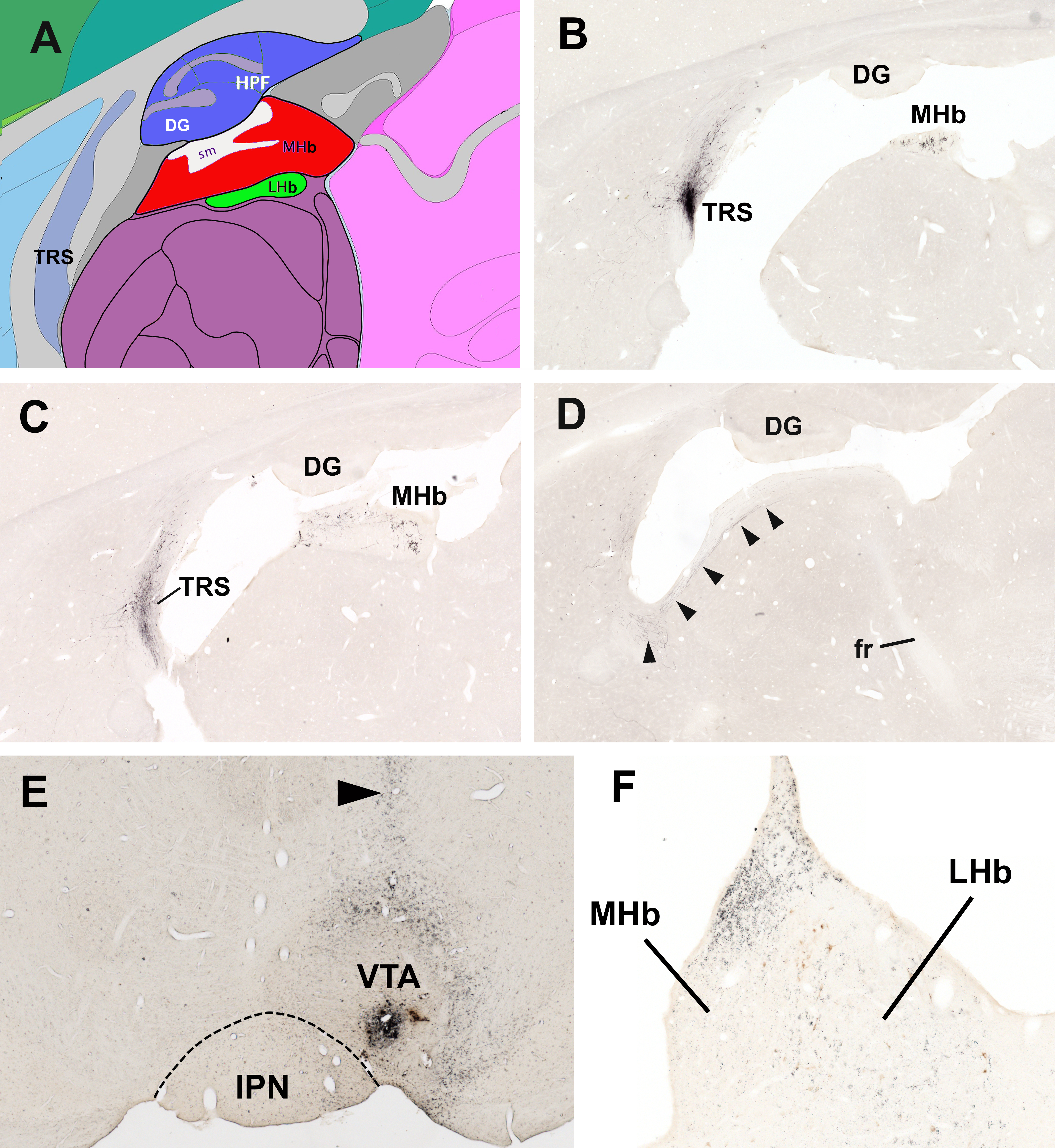

Supplement: Supplementary file 5 — Supplementary figure 4 [file 41398_2018_254_MOESM5_ESM.jpg]
